# One Pass Thalamic and Subthalamic Stimulation for Patients with Tremor-Dominant Idiopathic Parkinson Syndrome (OPINION): Protocol for a Randomized, Active-Controlled, Double-Blinded Pilot Trial

**DOI:** 10.2196/resprot.8341

**Published:** 2018-01-30

**Authors:** Peter Christoph Reinacher, Florian Amtage, Michel Rijntjes, Tobias Piroth, Thomas Prokop, Carolin Jenkner, Jürgen Kätzler, Volker Arnd Coenen

**Affiliations:** ^1^ Department of Stereotactic and Functional Neurosurgery Medical Center University of Freiburg Freiburg Germany; ^2^ Faculty of Medicine University of Freiburg Freiburg Germany; ^3^ Department of Neurology Medical Center University of Freiburg Freiburg Germany; ^4^ Clinical Trials Unit Freiburg Medical Center University of Freiburg Freiburg Germany

**Keywords:** Parkinson´s disease, Deep brain stimulation, Dentato-rubro-thalamic tract, Tremor, Ventral intermediate nucleus of thalamus (Vim), Subthalamic nucleus (STN)

## Abstract

**Background:**

Besides fluctuations, therapy refractory tremor is one of the main indications of deep brain stimulation (DBS) in patients with idiopathic Parkinson syndrome (IPS). Although thalamic DBS (ventral intermediate nucleus [Vim] of thalamus) has been shown to reduce tremor in 85-95% of patients, bradykinesia and rigidity often are not well controlled. The dentato-rubro-thalamic tract (DRT) that can directly be targeted with special diffusion tensor magnetic resonance imaging sequences has been shown as an efficient target for thalamic DBS. The subthalamic nucleus (STN) is typically chosen in younger patients as the target for dopamine-responsive motor symptoms. This study investigates a one-path thalamic (Vim/DRT) and subthalamic implantation of DBS electrodes and possibly a combined stimulation strategy for both target regions.

**Objective:**

This study investigates a one path thalamic (Vim/DRT) and subthalamic implantation of DBS electrodes and a possibly combined stimulation strategy for both target regions.

**Methods:**

This is a randomized, active-controlled, double-blinded (patient- and observer-blinded), monocentric trial with three treatments, three periods and six treatment sequences allocated according to a Williams design. Eighteen patients will undergo one-path thalamic (Vim/DRT) and STN implantation of DBS electrodes. After one month, a double-blinded and randomly-assigned stimulation of the thalamic target (Vim/DRT), the STN and a combined stimulation of both target regions will be performed for a period of three months each. The primary objective is to assess the quality of life obtained by the Parkinson’s Disease Questionnaire (39 items) for each stimulation modality. Secondary objectives include tremor reduction (obtained by the Fahn-Tolosa-Marin tremor rating scale, video recordings, the Unified Parkinson’s disease rating scale, and by tremor analysis), psychiatric assessment of patients, and to assess the safety of intervention.

**Results:**

At the moment, the recruitment is stopped and 12 patients have been randomized and treated. A futility analysis is being carried out by means of a conditional power analysis.

**Conclusions:**

The approach of the OPINION trial planned to make, for the first time, a direct comparison of the different stimulation conditions (Vim/DRT, compared to STN, compared to Vim/DRT+STN) in a homogeneous patient population and, furthermore, will allow for intraindividual comparison of each condition with the “quality of life” outcome parameter. We hypothesize that the combined stimulation of the STN and the thalamic (Vim/DRT) target will be superior with respect to the patients’ quality of life as compared to the singular stimulation of the individual target regions. If this holds true, this work might change the standardized treatment described in the previous section.

**Trial Registration:**

ClinicalTrials.gov: NCT02288468; https://clinicaltrials.gov/ct2/show/NCT02288468 (Archived by WebCite at http://www.webcitation.org/6wlKnt2pJ); and German Clinical Trials Register: DRKS00007526; https://www.drks.de/drks_ web/navigate.do?navigationId=trial.HTML&TRIAL_ID=DRKS00007526 (Archived by WebCite at http://www.webcitation.org/6wlKyXZZL).

## Introduction

Tremor is the most salient symptom of Parkinson’s disease (idiopathic Parkinson syndrome [IPS]). Other symptoms include bradykinesia, rigidity, and postural instability. As much as 75% of patients with IPS show resting tremor. Initially, tremor is typically unilateral and might be only visible during stressful situations. In the later stage of the disease it becomes bilateral.

The typical parkinsonian tremor is a resting tremor with or without an additional postural and/or kinetic tremor at the same tremor frequency. This is the most frequent tremor in Parkinson’s disease, termed type I tremor. Less than 10% of Parkinson patients develop a resting tremor and a postural tremor of different frequencies, termed type II tremor. A minority of IPS patients present a postural and/or kinetic tremor only, termed type III tremor.

Around 20% of patients with IPS will progress into candidates for Deep Brain Stimulation (DBS) in an advanced stage of the disease. DBS has become a standard treatment for the advanced stages of IPS [1,2,3]. Besides motor fluctuations, therapy refractory tremor (type I and to some extent type II) is one of the main indications of DBS in IPS [4].

First studies have shown that thalamic DBS, which targets the ventral intermediate nucleus (Vim) of thalamus, can effectively reduce Parkinson’s disease (PD) tremor (95%). In larger cohorts, this number was reduced to 85% favorable outcome [5,6]. It was also reported that the other symptoms of IPS are not favorably influenced with thalamic DBS and while tremor can be nicely controlled over the years, bradykinesia and rigidity are not well controlled under stimulation [6]. We have recently provided evidence that a fiber structure, the dentato-rubro-thalamic tract (DRT), that traverses the thalamic Vim region is a powerful target of thalamic Vim DBS. This structure can be directly targeted with the aid of diffusion tensor magnetic resonance imaging (DTI) sequences [7]. The use of subthalamic nucleus (STN) DBS shows effects on tremor but typically does not have dramatic initial effects on tremors like Vim-DBS does. However, STN DBS also reduces the other cardinal symptoms of IPS which Vim-DBS does not [8] [9]. There are anecdotal reports on pure STN stimulation's inability to effectively reduce tremor, hence the need to additionally stimulate the thalamic region. However, in recent years, STN stimulation has become the main treatment option for refractory IPS. The main indication for STN-DBS remains fluctuations in movement after long-standing dopaminergic medication [3]. Patients who suffer from tremor on top of these symptoms (equivalent type IPS) and who show some improvement with dopaminergic medication are likely to improve under STN-DBS [8]. However, different considerations apply for tremor-dominant IPS with therapy refractory tremor:

- In younger patients with tremor-dominant IPS, STN-DBS rather than thalamic (Vim/DRT) DBS appears to be the better option because early onset IPS is known to enter motor fluctuations in a later stage of the disease. These symptoms will likely respond to STN-DBS.
- Older patients who suffer from tremor-dominant IPS are less likely to develop motor fluctuations. Because of the higher complication rate of stimulation of the STN in this patient group [2], thalamic DBS is typically recommended.
- Especially older patients receiving thalamic DBS might—in a later stage of the disease—suffer from insufficient symptom control and these patients might benefit from additional STN surgery [10]. At this time, however, these patients might already be in a risk group for STN-DBS [2].

With this study, we will try to understand if patients with tremor-dominant IPS or patients with equivalent type IPS who perceive tremor to be their dominant symptom will benefit from a one-path thalamic and STN implantation of DBS electrodes and, possibly, a combined stimulation strategy for both target regions that is adjustable for the distinct target regions over time. Disease-related quality of life was chosen as the primary outcome. As it has been shown in the EARLYSTIM Study, this allows a global assessment of beneficial and adverse effects in a way that subjectively matters to the patient [1].

### Trial Purpose and Rationale

The proposed trial aims to investigate a combined approach to thalamic/subthalamic DBS for the treatment of patients with tremor-dominant IPS or patients with equivalent type IPS who perceive tremor to be their dominant symptom. As stated above, consensus exists for the application of STN versus thalamic (Vim/DRT) DBS in different age groups. While the younger age group appears to be clear candidate for STN-DBS, the older patient group (>60 years) remains to be problematic because of the above-mentioned reasons.

At the beginning of recruitment we could not detect any controlled study with an intrapatient comparison of thalamic versus STN DBS. We have performed a PubMed search (search as of 15 October 2014) with the search terms “DBS AND tremor AND Parkinson AND Vim AND STN”. In addition, we performed a search for (currently running) clinical trials on the World Health Organization International Clinical Trials Registry Portal (search as of 15 October 2014) with the search terms “DBS tremor parkinson” and “DBS tremor” and we did not find any other comparable trial recruiting and/or treating PD patients. There are case series only describing patients who had previous thalamus operation (Vim/DRT-DBS) and later received additional electrodes in the STN [10,11].

Recently we implanted bilateral octopolar DBS electrodes in the STN additionally traversing the DRT region via a parietal image-assisted approach in two patients allowing compassionate use of a combined stimulation of two tremor targets (STN and DRT) [12]. Both patients showed immediate and sustained improvement of their tremor and the symptoms of the bradykinetic syndrom, bilaterally.

## Methods

### Design

This is a randomized, active-controlled, double-blinded (patient- and observer-blinded), monocentric trial with three treatments, three periods, and six treatment sequences allocated according to a Williams design. The trial flow is illustrated in Multimedia Appendix 1. This monocentric study will be conducted at the Department of Stereotactic and Functional Neurosurgery in close collaboration with the Department of Neurology, both at the Freiburg University Medical Center, Germany.

The primary objective of this trial is to assess whether Quality of Life (QoL), obtained by the Parkinson’s Disease Questionnaire (PDQ-39) in Parkinson patients with combined Vim/DRT-DBS and STN-DBS is superior to treatment with either Vim/DRT-DBS or STN-DBS. This will be determined through assessment over a period of three months after implantation of Boston Scientific’s Vercise Deep Brain Stimulation System through the Vim/DRT into the STN using a posterior trajectory.

The secondary objectives are:

1. To show advantage of combined STN+Vim/DRT-DBS in tremor reduction in comparison to Vim/DRT-DBS or STN-DBS obtained by Fahn-Tolosa-Marin tremor rating scale (FTMTRS), video recording, the Unified Parkinson’s disease rating scale (UPDRS, part III, items 20 & 21), and by tremor analysis
2. To show superiority of combined STN+Vim/DRT-DBS in motor symptoms of Parkinson’s disease in comparison to Vim/DRT-DBS or STN-DBS obtained by Unified Parkinson’s disease rating scale (UPDRS, part III except items 20 & 21)
3. Psychiatric assessment of patients
4. To assess safety of intervention

### Participant Recruitment

Patients suffering from Parkinson’s disease who are referred to our department due to disabling medically resistant resting and/or postural tremor as their major complaint are informed about this study. Patients who give their informed consent are registered in the trial and undergo the screening procedures. Patients who gave their informed consent but do not undergo stereotactic surgery are regarded as screening failures.

Patients with Parkinson’s disease of both genders will be enrolled into this trial. No gender ratio has been stipulated. Inclusion and exclusion criteria are listen in Textbox 1.

A sample size of 18 male or female patients was calculated (details below). Recruitment will be stopped after the twelfth patient has completed his/her end of study visit (visit W40, 40 weeks after implantation of DBS system). A futility analysis will be carried out by means of a conditional power analysis. Based on the results of this analysis the study will either be continued or stopped.

### Study Events and Assessments

#### Screening

Screening assessments will be performed within 28 days prior to implantation. The patient will be admitted to hospital for this visit and inclusion and exclusion criteria are checked and validated. The complete pretherapeutic work-up includes a physical examination, consisting of a neurological examination and vital signs (including weight and height), medical history, demography, a pregnancy test in women of childbearing potential, Mattis Dementia Rating Scale, PDQ-39, UPDRS, FTMTRS with Video recording, CGI-I, tremor analysis, psychiatric assessment, PD medication, concomitant medication, L-Dopa equivalent dose (LED) and a cranial MRI.

#### Implantation of the Investigational Medical Device

The investigational medical device (IMD) for this study is Boston Scientific’s Vercise Deep Brain Stimulation System. This device is CE-marked but will not be used within the intended use for this clinical trial. The IMD will be implanted and programmed by the investigator. The investigator or authorized study personnel will document the implantation of each device in the respective forms. The patient will be admitted to hospital and the following assessments will be performed: (1) cranial computed tomography before implantation (planning); (2) cranial computed tomography after implantation (corroboration of electrode position); (3) concomitant medication; and (4) adverse events.

#### Imaging

Anatomical and diffusion tensor imaging is performed on a clinical 3 Tesla MRI system (Siemens Magnetom Trio Tim System 3T, Erlangen, Germany) a day before surgery under mild sedation with oral Lorazepam (1 - 2.5mg, Pfizer, Berlin, Germany) using a 12-channel head coil.

**Inclusion and exclusion criteria.**

Inclusion criteria

1. Male or female patients aged ≥ 35 and ≤ 75 years with a life expectancy of at least 5 years
2. Patients with Parkinson’s disease according to the criteria of the British Brain Bank as diagnosed by a neurologist specialized in movement disorders
3. Parkinson patients are included with a medical treatment resistant and disabling resting and/or postural tremor as their major complaint and with a less prominent or absent hypokinetic-rigid component of their disease.
4. Absence of postural instability (which could be aggravated under STN DBS)
5. Hoehn & Yahr stage 1-3. After stage 3 patients will show increased incidence of falling that can be aggravated by (typical) STN DBS
6. Disease duration for at least 2 years
  - and routine DAT-scan shows clear indication for Parkinsonism  
  - and atypical Parkinson syndromes are ruled out by routine glucose (FDG) PET  
7. PDQ-39 to be completed within 42 days prior to surgery
8. Written informed consent

Exclusion criteria

1. Major Depression with suicidal thoughts
2. Dementia (Mattis Dementia Rating Score ≤ 130)
3. Patients with lifetime primary psychotic disorder, schizophrenia, or schizoaffective disorder
4. Patients with acute psychosis as diagnosed by a psychiatrist
5. Nursing care at home
6. Unable to give written informed consent
7. Surgical contraindications like deformed or displaced or not discernable target region, scarring after brain disease (infarction), need for continuous anticoagulation that cannot be bridged in order to obtain normal coagulation
8. Patients with advanced stage cardiovascular disease
9. Patients under immunosuppressive or chemotherapy because of malignant disease
10. Patients who had previous intracranial surgery
11. Patients who are already under DBS therapy
12. Patients with aneurysm clips
13. Patients with cochlear implants
14. Simultaneous participation or previous participation within 30 days prior to start of screening in a clinical trial involving investigational medicinal product(s) or investigational medical device(s)
15. Medications that are likely to cause interactions in the opinion of the investigator
16. Known or persistent abuse of medication, drugs or alcohol
17. Persons who are in a relationship of dependence/employment with the sponsor or the investigator
18. Fertile women not using adequate contraceptive methods, such as female condoms, diaphragm or coil, each used in combination with spermicides; intra-uterine device; hormonal contraception in combination with a mechanical method of contraception
19. Current or planned pregnancy, nursing period
20. Contraindications according to device instructions or Investigator’s Brochure:
  - Diathermy: Shortwave, microwave, and/or therapeutic ultrasound diathermy. The energy generated by diathermy can be transferred to the Vercise DBS System, causing tissue damage at the contact site resulting in severe patient injury or death.
  - Magnetic Resonance Imaging (MRI): Patients implanted with the Vercise DBS System should not be subjected to MRI.
  - Patient incapability: Patients who are unable to properly operate the Remote Control and Charging System should not be implanted with the Vercise DBS System.
  - Poor surgical risks: The Vercise DBS System is not recommended for patients who‑—because of their primary disease or additional co-morbidities—are not likely to benefit from the DBS system implantation.

1. Anatomical sequences:
  - Three-dimensional (3D) magnetization-prepared rapid gradient-echo (MP-RAGE), repetition time (TR) 1 390 ms, echo time (TE) 2.15 ms, inversion time (TI) 800 ms, Flip angle 15°, voxel-size 1.0×1.0×1.0 mm^3^, acquisition time 3:15 min.
  - 3D T2 SPACE-sequence, TR 2 500 ms, TE 231 ms, echo train length 141, flip angle variable, voxel-size 1.0×1.0×1.0 mm^3^, acquisition-time 6:42 min.
2. Diffusion tensor imaging:
  - Single shot 2D SE EPI, TR 10 000 ms, TE 94 ms, Diffusion Values b=0 s/mm², b=1000 s/mm², diffusions-directions 61, slice count 69, voxel-size 2.0×2.0×2.0 mm^3^, acquisition time 11:40 min. Deformation correction of the EPI sequence according to Zaitsev et al. 2004 [13].

Deterministic Fiber tracking is performed on a Linux workstation using StealthViz DTI (Medtronic Navigation, Louisville, Colorado). An internal transfer procedure is used to fuse the line-graphic depiction of the DRT to the DICOM (Digital Imaging and Communications in Medicine) image that further serves for navigation purposes. With this procedure, the DRT becomes part of the stereotactic planning data. Fiber tracking of the cerebello-thalamo-cortical network (DRT) and surrounding structures (cortico-spinal tract) have been previously described [7,14-16].

#### Surgical procedure

After administration of standard antibiotic prophylaxis, a stereotactic frame (Leksell, Elekta, Stockholm, Sweden) was placed under local anesthesia. A Computed Tomography (CT) scan was performed and the image data were transferred to the planning workstation (Framelink 5.0, Medtronic SNT, Louisville, CO). The previously acquired MRI sequences and the DTI FT rendition of the DRT (as part of the stereotactic DICOM data) were coregistered with the stereotactic CT scan and the trajectories were planned taking into account mid-commissural point (MCP) coordinates (for STN we typically use: x=12; y-2, z=-4) and imaging of the targeted structures (DRT and STN). Where necessary, based on the imaging, the target was refined based on the direct visualization of the structures.

#### Description of the Operation

After administration of standard antibiotic prophylaxis, a stereotactic frame (Leksell, Elekta, Stockholm, Sweden) is placed under local anesthesia. A CT scan is performed and the image data are transferred to the planning workstation (Elekta, Stockholm, Sweden). The previously acquired MRI sequences are coregistered with the CT scan and the trajectories are planned, taking into account MCP coordinates and imaging of the targeted structures (Vim/DRT and STN).

**Figure 1:**
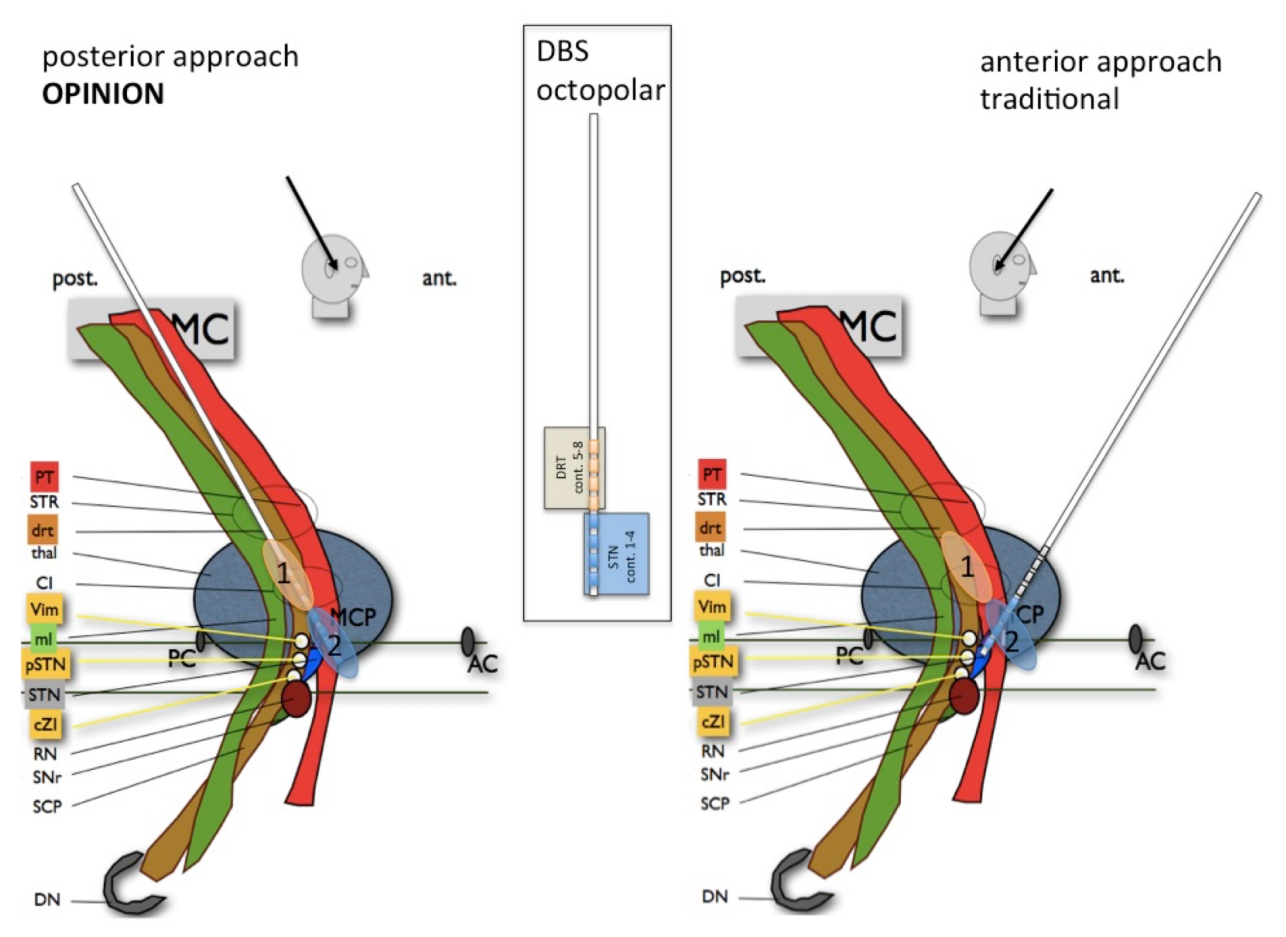
The proposed approach (left) and the traditional approach (right) to the subthalamic nucleus (STN) with dentato-rubro-thalamic tract (DRT) (1) and STN (2) stimulation sites. AC: anterior commissure; PC: posterior commissure; MCP: mid-commissural point; MC: primary motor cortex; CST: cortico-spinal tract; STP superior thalamic peduncle; DRT: dentato-rubro-thalamic tract; thal: thalamus; CI: internal capsule; Vim: ventral intermediate nucleus of thalamus stereotactic target (possibly this is the Vop ventralis oralis posterior nucleus), ml: medial lemniscus; pSTR: posterior subthalamic region; STN: subthalamic nucleus; cZI: caudal zona incerta; RN: red nucleus; SNr: substantia nigra; SCP: superior cerebellar peduncle; DN: dentate nucleus.  Figure from [12].

The bilateral DBS electrode implantation is performed under local anesthesia with the patient in a semisitting position. Using a microtargeting drive (MicroTargeting Star Drive M/E System, FHC Inc, Bowdoin, ME) a test electrode (Cosman Medical, Inc, Burlington, MA) is inserted through a parietal burr hole in the cranium (see Figure 1). Because of anticipated transventricular routes we do not to use sharp microelectrodes for microrecording, but instead rely on the imaging taking into account that the anterior, lateral, and STN (superior sensorimotor or dorsolateral STN) must be targeted [17]. Macrostimulation is performed to confirm a contralateral clinical benefit (tremor reduction at a low threshold for DRT, additional reduction of bradykinesia and rigidity more distally on the trajectory, in the STN) and to test for side effects (at a high threshold) in 2mm steps starting 4 mm above the individual target regions. The definitive DBS electrodes are then implanted under fluoroscopic control. An implantable pulse generator (Boston Scientific, Natick, MA) is implanted under general anesthesia during the same procedure. Postoperatively, all patients undergo a 3D CT scan to corroborate the final DBS electrode localization.

#### 1 Month Off Period

After implantation, the IMD will remain OFF for a period of 1 month. After implantation of a DBS electrode into the thalamic (Vim/DRT) or the subthalamic (STN) target area, most patients will experience a transient alleviation of their symptoms (rigidity, bradykinesia for STN, tremor for Vim/DRT). This is due to a microlesioning effect from electrode placement. This effect can last as short as days but can also last weeks. During this period programming is complicated because it is hard to differentiate between lesion and stimulation effects. Clinical practice shows that an interval of 3-4 weeks is sufficient between implantation and start of stimulation to get rid of most microlesioning effects. In this interval, medication can be expected to be kept unchanged because of the characteristics of the study population regarded here.

### Week 4 (Baseline, treatment start)

Within 7 days prior to treatment start the patient will be randomized.

### Assessments at visit Week 4

The patient will be admitted to hospital for this visit. The following assessments have to be performed: PDQ-39, UPDRS, FTMTRS, CGI-I, video recording, tremor analysis, psychiatric assessment, vital signs (including weight), PD medication, concomitant medication, LED, and adverse events.

### Treatment start

Stimulation procedure is conducted by an unblinded investigator, who is not involved in data acquisition. All stimulation contacts will first be checked for impedance as an indicator for cable break, short-circuit or other device-related complications prior to stimulation. Afterwards all eight contacts will be tested for clinical effect on both tremor and hypokinetic-rigid symptoms (rigidity, bradykinesia). Thresholds for side effects and side effects will be evaluated. Therapeutic effects and adverse effects (and between them the therapeutic window) will be noted in a standardized protocol. Stimulation settings will then follow the randomization of the area which has to be stimulated (Vim/DRT, STN or, Vim/DRT-STN). Stimulation parameters are set empirically on the estimation of the investigator based on the testing phase of electrode contacts, with tremor being the primary target symptom.

### Week 6, 8 and 12

These visits can be performed either via telephone or at the clinical site. The following assessments have to be performed: PD medication, concomitant medication, LED, and adverse Events.

### Week 16 (first Treatment Switch)

If clinically indicated, the patient will be admitted to hospital for this visit. The following assessments have to be performed: PDQ-39, UPDRS, FTMTRS, CGI-I, video recording, tremor analysis, psychiatric assessment, vital signs (including weight), PD medication, concomitant medication, LED, and adverse events.

Stimulation settings follow the randomization of the area, which has to be stimulated (Vim/DRT, STN or Vim/DRT+STN). Stimulation parameters are set empirically on the estimation of the investigator based on the testing phase of electrode contacts at baseline, with tremor being the primary target symptom.

### Week 18, 20 and 24

These visits will be performed in the same way as visits in week 6, 8 and 12.

### Week 28 (second Treatment Switch)

This visit will be performed in the same way as the week 16 visit.

### Week 30, 32 and 36

These visits will be performed in the same way as visits in weeks 6, 8, and 12.

### Week 40: End of study

If clinically indicated, the patient will be admitted to hospital for this visit. Assessments performed will be the same as those in the week 16 visit.

### Discontinuation criteria

The coordinating investigator is under obligation to monitor the progress of the clinical trial with regard to safety-relevant developments and, if necessary, initiate the premature termination of a treatment arm or the entire clinical trial.

#### Premature Termination of One of the Treatment Arms or the Entire Trial

A treatment arm or the entire clinical trial must be terminated prematurely if:

- The benefit-risk ratio for the patient changes markedly and/or indications arise that the trial patients' safety is no longer guaranteed, defined as: after surgical treatment of the sixth patient, two or more patients experienced severe intra-cranial hemorrhage or ischemia (as diagnosed with computed tomography) or infection and/or severe neurological deterioration (hemiparesis persisting over 24 hours). In this case, recruitment will be stopped and the Data Monitoring Committee (DMC) will discuss continuation of the trial. Bleeding rate is known to be between 1-3% and approx. 0.78% of patients experience a clinically significant bleeding [18] (eg, life changing complications because of persisting disabilities). Therefore, it seems appropriate to temporarily hold the trial if two or more patients out of the first six implanted subjects experience the aforementioned severe complications.
- Following recommendation of the DMC (eg, after futility analysis) the coordinating investigator considers that the termination of the trial is necessary
- The question(s) addressed in the trial can be clearly answered on the basis of an interim analysis
- The questions(s) addressed in the trial can be clearly answered on the basis of results of another trial on the same subject
- An insufficient recruitment rate makes a successful conclusion of the clinical trial appear impossible (eg, less than 3 patients are recruited per year)

#### Premature Discontinuation of Deep Brain Stimulation

DBS therapy of a patient will be terminated prematurely in the following cases:

- Adverse events (including intercurrent illnesses) which preclude further treatment with the IMD or make further participation in the clinical trial inadvisable because the informational value of the trial results is impaired
- Premature termination of the trial treatment is considered to be medically indicated, eg, because it is subsequently found that inclusion/exclusion criteria were violated
- Continuation of the trial treatment is unacceptable when the risks outweigh the benefits. This is the case if stimulation treatment induces unstable gait and falls or unbearable side effects like severe dyskinesia.
- Pregnancy
- Significant violations of the trial protocol or lack of compliance on the part of the patient
- Logistical reasons (patient changes his/her doctor or hospital or moves to another location)

Follow-up visits will be performed as far as possible.

#### Premature Termination of Trial Participation

The trial patient can withdraw his/her consent at any time, without having to give reasons, and have his/her entire trial participation terminated prematurely. If a patient withdraws informed consent no further follow-up is possible.

### Biostatistical Planning and Analysis

Before the start of the final analysis a detailed statistical analysis plan will be prepared. This will be completed during the “blind review” of the data, at the latest. This blind review, ie, a checking and assessment of the data, will be performed before the futility analysis and the planned follow-up period without looking at the randomized treatment for each patient. If the statistical analysis plan contains any changes to the analyses outlined in the trial protocol, they will be marked as such, and reasons for amendments will be given.

All statistical programming for analysis will be performed with the Statistical Analysis System.

#### Sample Size Calculation

Based on the standard error of PDQ-39 total score in the EARLYSTIM trial [1], we anticipate a within-person standard deviation of about 14.4 score points for the difference between two treatments. If 18 patients (3x6) are allocated to each of the 6 sequences, a two-sided t-test (analysis of variance for difference of means in crossover designs) at significance level 5% has 80% power to detect a difference if the true mean difference between STN+Vim/DRT-DBS and STN-DBS (or Vim/DRT-DBS) is 10.2 points (effect size: 0.71; nQuery Advisor version 7.0).

#### Randomization

Fax randomization will be performed within 7 days prior to treatment start. The patient identification code assigned for the trial will be entered on the randomization form and the fully completed form will then be faxed to the Central Randomization Office of the Clinical Trials Unit. Patients will be randomized to 6 treatment sequences. The block-lengths will be documented separately and will not be disclosed. The randomization code will be generated by the Clinical Trials Unit using the following procedure to ensure that treatment assignment is unbiased and concealed from patients and investigative staff. Patients will be randomized to 6 treatment sequences according to a Williams design. The randomization code will be produced by validated programs based on the Statistical Analysis System.

#### Blinding

Participating patients and (external) observers and raters are blinded. Since stimulation procedure (eg, start of treatment, treatment switch, adjusting of stimulation parameters/settings) is conducted by an unblinded investigator who is not involved in data acquisition, blinding will be maintained for patients and for observers and raters.

#### Description of the Primary Efficacy Analysis and Population

Analysis of the primary endpoint will be done by intention to treat in a linear mixed model with baseline score, treatment, period and sequence included as fixed effects [18], and within-patient correlation modelled by a compound symmetry covariance matrix to account for the random subject effect. The sequence effect will be dropped if nonsignificant at the 5%-level. In the final model, treatment comparisons will be based on contrasts estimated by least-squares means with two-sided 95% confidence intervals. For confirmatory analysis, a closed test procedure will be applied: First, the null-hypothesis of equal means in the three arms will be tested at a significance level of 5%. Only if it can be rejected will the three pairwise treatment comparisons be carried out in a confirmatory fashion. This multiple test procedure assures control of the multiple type I error rate of 5%. In addition, all fixed effects will be tested descriptively at the two-sided 5%-level. Recruitment will be stopped after 12 patients have been randomized and treated. Then, a futility analysis will be carried out by means of a conditional power analysis. The conditional probability to attain a significant result for STN+Vim/DRT-DBS versus STN-DBS and Vim/DRT-DBS, respectively, after recruitment of another 6 patients, given the results of the first 12 patients, will be estimated. Three scenarios will be considered: (1) the effect size for the 6 patients to follow will be estimated from the results of the first 12 patients, (2) the upper (optimistic) limits of the 95% confidence intervals for the treatment effects estimated from the first 12 patients will be used, and (3) the treatment effect for the 6 patients to follow will be assumed to be 10.2 points as anticipated in the sample size calculation. The optional possibility to stop the trial prematurely for futility after this interim analysis does not inflate the type one error rate. If the conditional power is below 30% in all three chosen scenarios the trial will be stopped. If the conditional power of any of the three scenarios is between 30-50%, the DMC will decide on the continuance of the trial. If the conditional power of any of the three scenarios is above 50% the trial will be continued.

### Ethics and Dissemination

An adequate subject insurance contract has been taken out. The study protocol has been approved by the independent Ethics Committee of the University of Freiburg (reference number EK 38/15) and by the Federal Institute for Drugs and Medical Devices (reference number 94.1.04 - 5660 – 9558). The study will be conducted in accordance with the ethical principles of the Declaration of Helsinki, the DIN EN ISO 14155, and applicable regulatory requirements (eg, German Medical Devices Act, Ordinance on Clinical Trials with Medical Devices). The OPINION trial has been registered in the publicly available registries: ClinicalTrials.gov (NCT02288468) and German Clinical Trials Register (DRKS00007526).

#### Informed consent

Before enrolment in the clinical trial, the patient will be informed that participation in the clinical trial is voluntary and that he/she may withdraw from the clinical trial at any time without having to give reasons and without penalty or loss of benefits to which the patient is otherwise entitled.

The treating physician will provide the patient with information about the treatment methods to be compared and the possible risks involved. At the same time, the nature, significance, implications, expected benefits and potential risks of the clinical trial and alternative treatments will be explained to the patient. During the informed consent discussion, the patient will also be informed about the insurance cover that exists and the insured's obligations. The patient will be given ample time and opportunity to obtain answers to any open questions. All questions relating to the clinical trial should be answered to the satisfaction of the patient. In addition, the patient will be given a patient information sheet which contains all the important information in writing. The patient's written consent must be obtained before any trial-specific tests/treatments. For this purpose, the written consent form will be personally dated and signed by the trial patient and the investigator conducting the informed consent discussion.

#### Safety

Adverse Events will be documented in the case report form and in the patient’s medical chart (source documents). Serious Adverse Events will be reported according to the provisions set forth in the German Medical Devices Safety Plan Ordinance.

### Data Monitoring Committee

The DMC will consist of the coordinating investigator and the unblinded investigators. As stated above, the trial will be stopped if the conditional power is below 30% in all three scenarios; the trial will continue if the conditional power of any of the three scenarios is above 50%. If the conditional power is between 30–50% the DMC will decide on the continuance of the trial. For this purpose, the DMC will receive unblinded trial data and will discuss whether or not continuation of the trial is ethically justified.

The DMC will also receive data on severe intracranial hemorrhages or ischemias or infections and/or severe neurological deteriorations. In case of higher occurrence rates than expected, the DMC will discuss whether the trial should be stopped prematurely.

## Results

Recruitment to the OPINION trial opened in July 2015 and will close in September 2019. At the time of manuscript submission, the recruitment is stopped; 12 patients have been randomized and treated and a futility analysis is being carried out by means of a conditional power analysis.

## Discussion

The approach planned to investigate in the OPINION trial will, for the first time, allow for the direct comparison of the different stimulation conditions (Vim/DRT, STN, and Vim/DRT+STN) in a homogeneous patient population and will furthermore allow an intraindividual comparison of each condition with the outcome parameter “quality of life”. We hypothesize that the combined stimulation of the STN and the thalamic (Vim/DRT) target will be superior with respect to the patients’ quality of life as compared to the singular stimulation of the individual target regions. If this holds true, this work might change the standardized treatment described in the previous section.

## Appendix

Multimedia Appendix 1 Trial flow of the randomized, active-controlled, double blinded (patient and observer blinded), monocentric trial with three treatments, three periods and six treatment sequences allocated according to a Williams design.

Multimedia Appendix 2 CONSORT-EHEALTH (V 1.6.1).

## Abbreviations

CT: Computed tomography
CGI-I: Clinical Global Impression – Global Improvement Scale
DBS: Deep brain stimulation
DRT: Dentato-rubro-thalamic tract
DICOM: Digital Imaging and Communications in Medicine
DTI FT: Diffusion tensor imaging fiber tractography
DTI: Diffusion tensor magnetic resonance imaging
FTMTRS: Fahn-Tolosa-Marin tremor rating scale
IMD: Investigational medical device
IPS: Idiopathic Parkinson syndrome
LED: L-Dopa equivalent dose
MCP: Mid-commissural point
MRI: Magnetic Resonance Imaging
PD: Parkinson’s disease
PDQ-39: Parkinson’s Disease Questionnaire -39 items
QoL: Quality of life
STN: Subthalamic nucleus
TE: Echo time
TR: Repetition time
UPDRA: Unified Parkinson’s disease rating scale
Vim: Ventral intermediate nucleus
