# Supplementary figures and images for: One Pass Thalamic and Subthalamic Stimulation for Patients with Tremor-Dominant Idiopathic Parkinson Syndrome (OPINION): Protocol for a Randomized, Active-Controlled, Double-Blinded Pilot Trial
Source: JMIR Res Protoc. 2018 Jan 30;7(1):e36. doi: 10.2196/resprot.8341 (PMC5811645; doi:10.2196/resprot.8341)

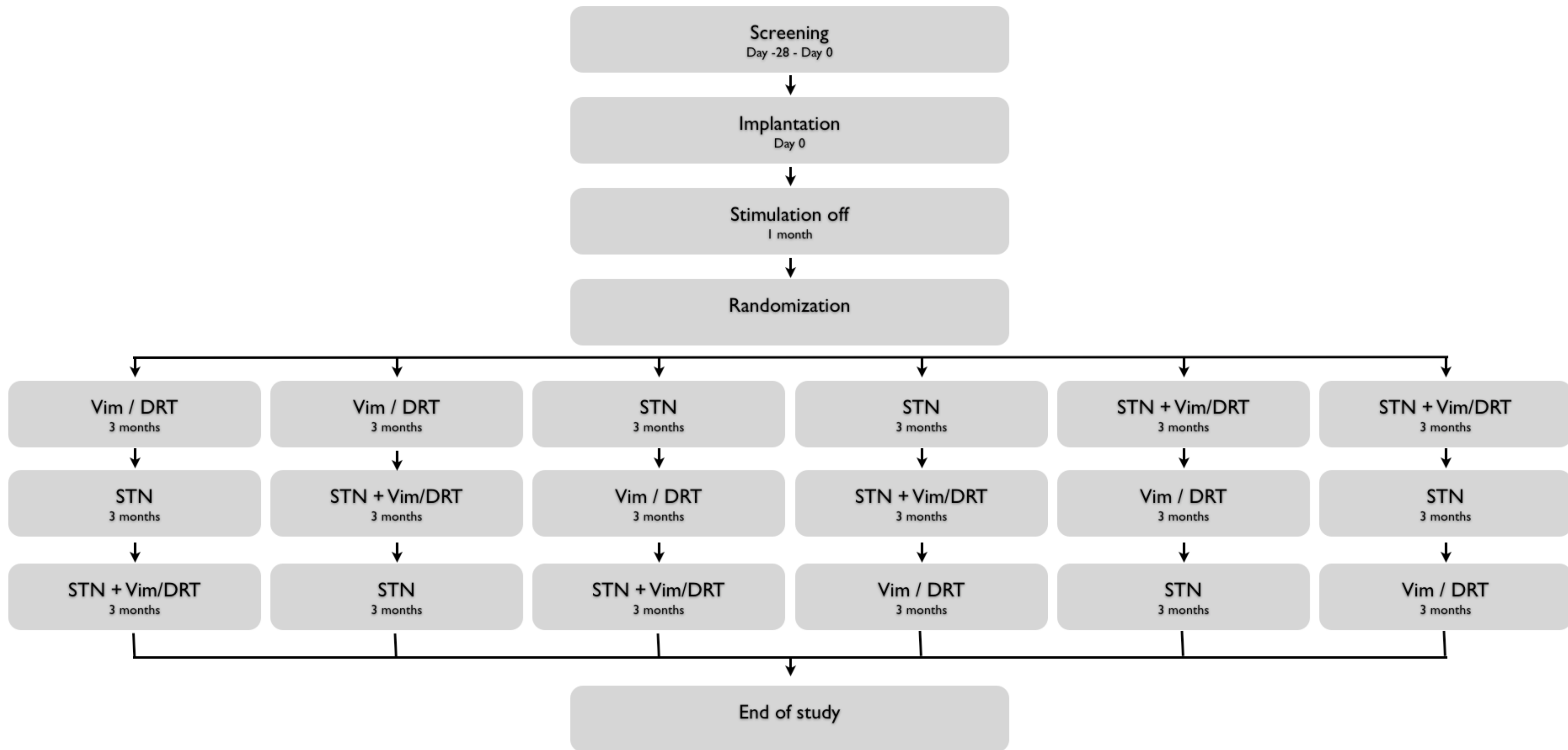

Supplement: Multimedia Appendix 1 [file resprot_v7i1e36_app1.pdf]
